# Novel compound heterozygous frameshift variants in *WDR81* associated with congenital hydrocephalus 3 with brain anomalies: First Chinese prenatal case confirms *WDR81* involvement

**DOI:** 10.1002/mgg3.1624

**Published:** 2021-03-16

**Authors:** Jiasun Su, Weiliang Lu, Mengting Li, Qiang Zhang, Fei Chen, Shang Yi, Qi Yang, Sheng Yi, Xunzhao Zhou, Limei Huang, Yiping Shen, Jingsi Luo, Zailong Qin

**Affiliations:** ^1^ Guangxi Health Commission Key Laboratory of Precise Diagnosis and Treatment of Genetic Diseases Maternal and Child Health Hospital of Guangxi Zhuang Autonomous Region Nanning China; ^2^ Genetic and Metabolic Central Laboratory Guangxi Birth Defects Research and Prevention Institute Nanning China; ^3^ Department of Genetics Harvard Medical School Boston MA USA

**Keywords:** compound heterozygous frameshift variants, congenital hydrocephalus 3 with brain anomalies, prenatal, *WDR81*

## Abstract

**Background:**

Congenital hydrocephalus‐3 with brain anomalies (HYC3, MIM 617967) is a rare form of congenital hydrocephalus characterized by severe hydrocephalus and cerebellar abnormalities, the onset of the disease occurs in utero even resulting in fetal death. A very limited spectrum of *WDR81* pathogenic variants had been reported in three unrelated families with HYC3. This study aims at presenting novel compound heterozygous frameshift variants in *WDR81* in a Chinese fetus.

**Methods:**

Whole‐exome sequencing (WES) was performed for a fetus with multiple congenital anomalies including sever hydrocephalus, cleft lip and palate, hydrops fetalis, hepatomegaly, and cerebellar hypoplasia. Sanger sequencing was performed to confirm the origin of the variants subsequently. Variants classification was based on the American College of Medical Genetics and Genomics/Association for Molecular Pathology (ACMG/AMP) guidelines.

**Results:**

Two novel heterozygous variants c.146_147insG (p.Thr52fs) and c.673delC (p.Leu225fs) in *WDR81* were identified. Sanger sequencing revealed that the c.146_147insG mutation was maternal origin and the c.673delC mutation was paternal origin. Both variants were pathogenic according to the ACMG/AMP guidelines.

**Conclusion:**

The present study expands the mutation spectrum of *WDR81* and help further define the genotype–phenotype correlations of HYC3. *WDR81*‐related HYC3 were highly clinical heterogeneity. We suggested that fetal hydrocephalus with extracerebral manifestations may be suggestive of *WDR81* deficiency and WES is effective for achieving a conclusive diagnosis for disorder.

## Graphical abstract

We identified a fetus with a Novel compound heterozygous frameshift variants in WDR81(c.146_147insG (p.Thr52fs) and c.673delC (p.Leu225fs)) presented with the main Congenital hydrocephalus 3 with brain anomalies (HYC3). This was a first Chinese prenatal case with HYC3 phenotype carried the WDR81 pathogenic variants detected by whole‐exon sequencing.

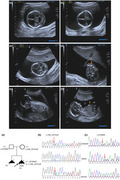

## 1 INTRODUCTION

Congenital hydrocephalus is a clinically and genetically heterogeneous disorder in fetuses, with an estimated incidence of 4.65 per 1,000 newborns in European regions (Garne et al., 2010), generally characterized as abnormal cerebrospinal fluid circulation which result in progressive expansion of the cerebral ventricles. It has been classified into syndromic and nonsyndromic forms based on whether extra clinical signs are present (Tully & Dobyns, 2014). Nonsyndromic hydrocephalus includes the classical X‐linked type associated with mutations in *L1CAM* (MIM 307000) and autosomal recessive hydrocephalus related to the gene *CCDC88C* (HYC1, MIM 236600), *MPDZ* (HYC2, MIM 615219), and *WDR81* (HYC3, MIM 617967). The HYC3 is a rare form of congenital hydrocephalus, it was initially identified in two unrelated consanguineous Saudi families, patients with HYC3 presented severe hydrocephalus and cerebellar abnormalities, other symptoms including macrocephaly, dysmorphic facial features, and polyhydramnios. This disease with an onset in‐utero and exhibit as a lethal form of congenital hydrocephalus (Shaheen et al., 2017).

*WDR81* is located at 17p13.3, its longest isoform encompassing 10 exons encoding 1941 amino acids, compose with an N‐terminal Beige and Chediak‐Higashi (BEACH) domain, a major facilitator superfamily (MFS) domain and a six WD40 beta‐propeller repeats on C‐terminus. Mutations in *WDR81* are associated neurological disorders including CAMRQ2 (cerebellar ataxia, cognitive disability, and disequilibrium, MIM 610185), sever microcephaly and HYC3. Only three HYC3 cases with *WDR81* mutations had been reported recently (Cappuccio et al., 2017; Shaheen et al., 2017). Thus, the variant spectrum of *WDR81* and the phenotype association with HYC3 are still very limited.

Herein, we present two novel compound heterozygous frameshift variants in *WDR81* that associated with autosomal recessive HYC3 in a Chinese fetus for the first time, which could help improve the recognition of this syndrome and the genetic implications for families identified in the future.

## 2 MATERIALS AND METHODS

### 2.1 Ethical compliance

This study was approved by the Medical Ethics Committee of the Maternal and Child Health Hospital of Guangxi Autonomous Region. Written informed consent was obtained from the family for publication of their pertinent images included in this paper.

### 2.2 DNA extraction

The genomic DNA was extracted from the parents blood and their fetus tissue using Lab‐Aid DNA kit (Zeesan Biotech Co, Ltd). DNA concentration and quality were determined by Q‐bit (Thermo Fisher Scientific). Approximately 200 ng of genomic DNA from each sample was randomly fragmented into 150‐ to 200‐basepair length by ultrasonicator (M220, Covaris).

### 2.3 Whole‐exome sequencing

Whole‐exome sequencing (WES) was performed by parent's request. DNA library was constructed by Agilent SureSelect Human Exon V5 kit (Agilent Technologies) according to the manufacturer's protocols. Sequencing was processed on Illumina HiSeq X Ten System (Illumina, Inc) based on the manufacturer's protocols. The sequencing reads were mapped to the Genome Reference Consortium Human genome build 37 (GRCh37). The Genome Analysis Toolkit (GATK) was used for variant calling. Candidate single nucleotide variants (SNVs) and insertion‐deletions (indels) were saved in VCF files and uploaded to the online variation annotation tool TGex (https://tgex.genecards.cn/#/) for further filtering and prioritizing. Common variants were filtered based on the frequencies in the Exome Aggregation Consortium (ExAC) (http://exac.broadinstitute.org), the Exome Sequencing Project (https://esp.gs.washington.edu), the 1000G (http://www.1000genomes.org), genomAD (http://gnomad.broadinstitute.org/) and our local database. The variant pathogenicity was assessed according to the American College of Medical Genetics and Genomics/Association for Molecular Pathology (ACMG/AMP) guidelines (Richards et al., 2015).

### 2.4 *WDR81* variants validation

Sanger sequencing was performed for the validation of candidate variants identified by WES. The primers for the amplification of targeted regions of the *WDR81* gene (NM_001163809.1) were designed by the Prime Z (http://grch37.genepipe.ncgm.sinica.edu.tw/primerz/beginDesign.do), the specificity and reliability of primers were evaluated by online UCSC In‐Silico PCR. The primers designed for the candidate variant (c.146_147insG) were listed as follows: forward (5′‐3′): CCGCCAAGCCCAGACAT, and reverse (5′‐3′): AAGGGTGTACCACATACAGCATC. Primers designed for candidate variant (c.673delC) were as follows: forward (5′‐3′): CCCAGAATTATCGCAACCTG and reverse (5′‐3′): CAGCTGCATGAGGTAGTGGA. The primers were synthesized by Invitrogen Biotechnology, Shanghai, China. Polymerase chain reactions (PCR) was performed (Takara Biotechnology) and products were sequenced by Thermo Fisher Scientific, Guangzhou, China. Sequences alignment were performed by SnapGene version 2.3.2.

## 3 RESULTS

### 3.1 Clinical information and ultrasound findings

**FIGURE 1:**
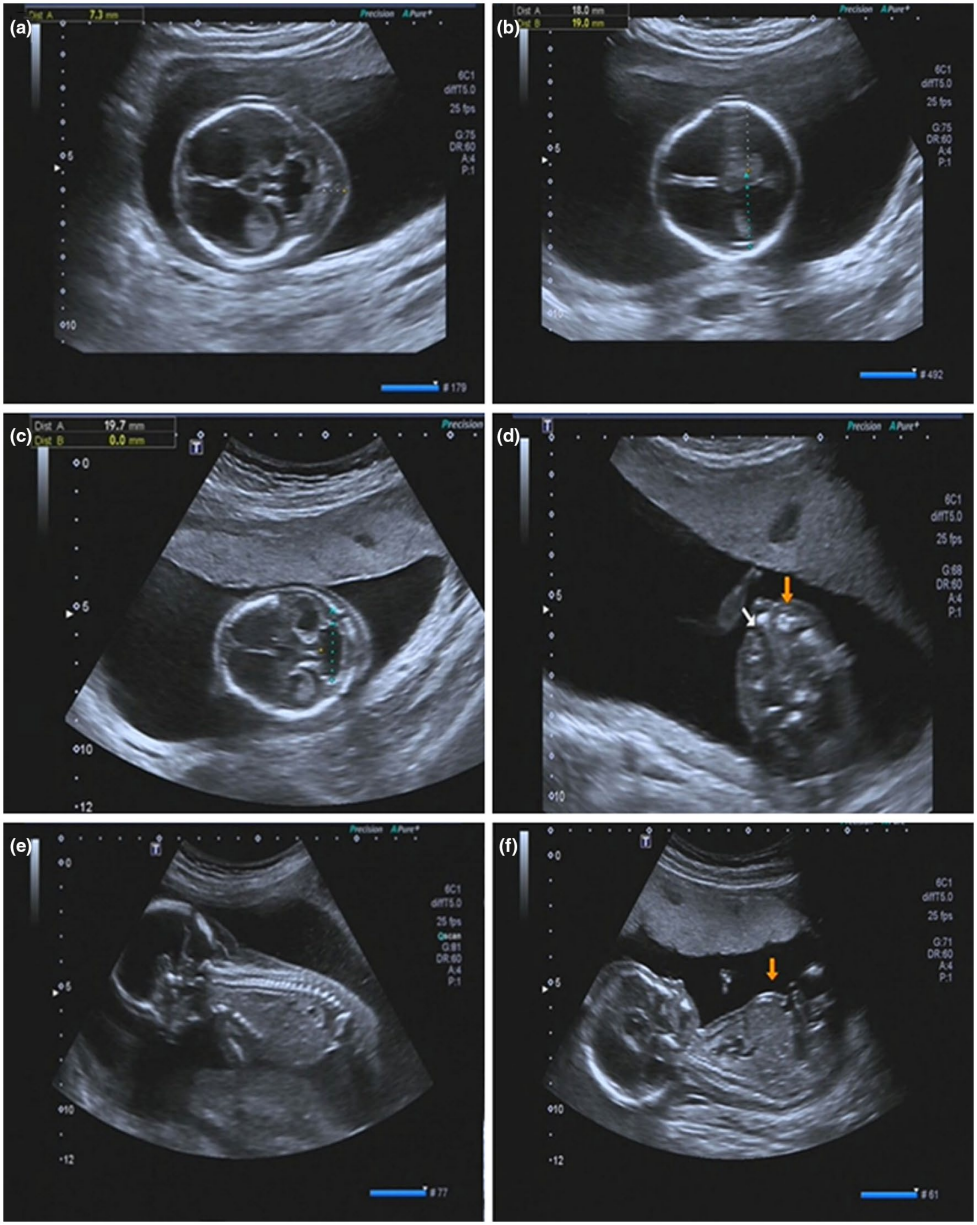
Ultrasound images of the fetus at 16 gestation weeks. (a) ultrasonographic examination indicated fetal cerebral ventriculomegaly, increased nuchal fold thickness (7.3 mm), suspected hydrocephalus and suggesting hydrocephalus (b). (c) measurement of transverse cerebellar diameter (TCD), and the ultrasonographic images suggesting the cerebellar dysplasia, cleft lip and palate (d), fetus hydrops (e) and bulging abdomen (f)

A 30‐year‐old, gravida 2, para 0 woman was referred for genetic counseling at 16 weeks of gestation because of fetal abnormalities on prenatal ultrasound. Her husband was 32 years of age and healthy. The couple had a previous history of fetal hydrocephalus diagnosed with multiple congenital anomalies, including increased nuchal fold thickness (INF), hydrocephalus, and ventricular septal defect (VSD) and the family terminated the pregnancy (TOP) directly without performing any further molecular tests by consideration of the poor prognosis. Similar findings were detected on the second trimester ultrasound examination for this pregnancy at 16 gestation weeks revealed the fetal hydrocephalus, cleft lip and palate, INF, hydrops fetalis, hepatomegaly and cerebellar hypoplasia (Figure 1). Chorionic villus sampling had performed for this pregnancy and chromosome microarray analysis (CMA), conventional karyotypes were negative, the woman opted for TOP in another hospital.

### 3.2 Variants analysis and validation

**FIGURE 2:**
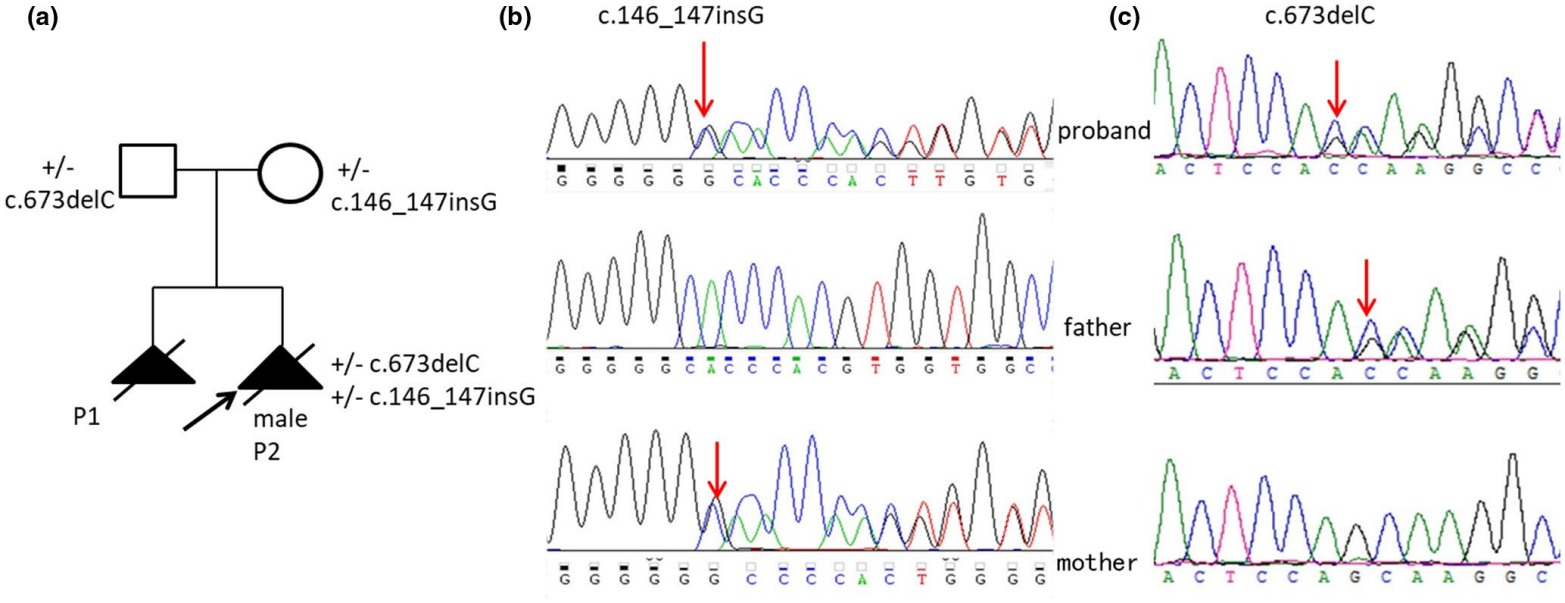
Identification of the compound heterozygous frameshift variants in *WDR81*. (a) Pedigree of family. P1 presented multiple congenital anomalies including hydrocephalus, INF, VSD and the family opted TOP directly without any further tests. Two heterozygous mutations of *WDR81*, NM_001163809.1: c.146_147insG and c.673delC in exon 1 in the proband P2 were confirmed by Sanger sequencing, the insertion variant c.146_147insG was inherited from the mother (b) and the deleterious variants c.673delC was inherited from the father (c)

Genomic DNA was obtained from the fetus for WES, a total of >99% reads were mapped to genomic targets with 20X coverage for >96% of capture regions. A total of 26,478 SNV or indel variants were identified in coding regions and splice sites. After removing synonymous SNPs and polymorphisms in dbSNP and 1000 Genomes, and removing the variants with a minor allele frequency (MAF)>3% in gnomAD, ESP, 1000G and our internal database, there were 1009 variants remaining with a MAF <0.01, furthermore, the neutral and benign variants were also excluded according to ClinVar database. Clinical features included fetal hydrocephalus, cleft lip and palate, hydrops fetalis, hepatomegaly, and cerebellar hypoplasia were regarded as filtration parameters for variant screening, seven candidate variants matched with known phenotypes in six genes (*WDR81*, *CCDC88C*, *TRMU*, *COL4A1*, *FGFR3*, *POMGNT2*) were extracted. Two novel heterozygous variants in *WDR81* (NM_001163809.1), c.146_147insG (p. Thr52fs) and c.673delC (p. Leu225fs) in exon 1 were identified. Sanger validation showed that c.146_147insG mutation was maternal origin and the c.673delC mutation was paternal origin (Figure 2). According to the ACMG/AMP guidelines (Richards et al., 2015), c.146_147insG (p. Thr52fs) was classified as pathogenic (PVS1, PM2, PP4: 1 pathogenic very strong evidence, 1 pathogenic moderate evidence and 1 pathogenic supporting evidence) and c.673delC (p. Leu225fs) was also classified as pathogenic (PVS1, PM2, PM3, PP4: 1 pathogenic very strong evidence, 2 pathogenic moderate evidence and 1 pathogenic supporting evidence).

## 4 DISCUSSION

*WDR81*‐related neurological diseases including autosomal recessive CAMRQ2, microcephaly and HYC3. Due to the overlapped clinical features between CAMRQ2 and HYC3 cases such as the brain atrophy or hypoplastic cerebellum, the specific diagnosis was difficult to define, especially in prenatal. To clarify the fetal disease, we first analyzed all the reported patients with *WDR81*‐related neurological phenotype and the summary were described in Table 1. Overall, cerebellar anomalies were commonly presented in all patients especially hydrocephalus in HYC3. In our case, the fetus exhibited sever hydrocephalus and cerebellar hypoplasia, meeting the core phenotype of HYC3. In addition, extracerebral anomalies included cleft lip and palate, hydrops fetalis and INF that had not been described in literature before were also found with this fetus from our observation, hydrocephalus and the disease onset time may be the characteristic indicator for HYC3, the onset of CAMRQ2 was in infancy and the onset of HYC3 was in‐utero even most patient die in utero or shortly after birth, other non‐specific signs including macrocephaly, ventricles abnormalities, and dysmorphic facial features also could be found in prenatal (Table 1). Our findings supported the notion that *WDR81*‐related neurological diseases were highly clinical heterogeneity, extracerebral manifestations suggesting the multifunctional properties of *WDR81*. More cases would be need for further investigation.

**TABLE 1:** Clinical features of patients with *WDR81* variants reported previously and in this study.

Disease	Gender	Age	Variants (NM_001163809.1)	Exon	Phenotype	References
HYC3	NA	28GW	c.845G>A (p. Gly282Glu)	1	Severe hydrocephalus, Hypoplastic cerebellum	Shaheen et al. (2017)
HYC3	m	29GW	c.3286C>T (p. Gln1096*)	1	Polyhydramnios, Hydranencephaly, Absent cerebellum	
HYC3	f	17 years	c.3693G>A (p. Trp1231*), c.5464C>T (p. Arg1822*)	2,9	Facial dysmorphism (ocular proptosis, thin upper lip, bulbous nose, mid‐face hypoplasia, and macroglossia), Absent corpus callosum, Enlarged third ventricle, Cerebellar hypoplasia, Extremely thin frontal lobes, Microcephaly	Cappuccio et al. (2017)
CAMRQ2	3f/2 m	28 yrs	c.2567C>T (p. Pro856Leu)	1	Short stature, Intellectual disability, Cerebellar ataxia, Ophthalmoplegia, Normal/brain atrophy, Thin corpus callosum, Cerebellar atrophy, Vermis midline cleft	Gulsuner et al. (2011)
CAMRQ2	NA	Neonatal	c.845G>A (p. Gly282Glu)	1	Neonatal death, Severe hydranencephaly, Severe cerebellar hypoplasia	Alazami et al. (2015)
CAMRQ2	f	3 yrs	c.3,997C>T (p. Arg1,333*)	4	Global developmental delay, Speech impairment, Cerebellar ataxia, Mild cerebellar atrophy	(Komara et al., 2016)
Severe microcephaly	m	22–27 M	c.1882C>T (p. Gln628*), c.3713C>G (p. Pro1238Arg)	1,2	Extreme microcephaly, Spastic, tetraplegia, Generalized, Dyskinesia, Nystagmus neonatal, Lissencephaly, Thin corpus callosum, Enlarged ventricles and Subarachnoid space	Cavallin et al. (2017)
Microcephaly	f	25GW	c.2834_2837delTGTT (p. Phe946Serfs*17), c.5464C>T (p. Arg1822*)	1,9	Relatively microcephaly, Delayed primary gyration, Corpus callosum agenesis, Severe brainstem hypoplasia, Cerebellum hypoplasia	
Severe microcephaly	f	14.5–22 yrs	c.1582C>T (p. His528 Tyr), c.4036_4041dup (p. Val1346_Thr1347dup)	1,4	Extreme microcephaly, Spastic tetraplegia, Generalized dyskinesia, Nystagmus, Gyral simplification, Thin corpus callosum, Cerebellar atrophy, Periventricular gliosis	
Severe microcephaly	m	4–13 yrs	c.1735G>A (p. Gly579Arg), c.1358 dup (p. Tyr453*)	1	Extreme microcephaly, Spastic tetraplegia, Infantile spasms, Dystonia, Nystagmus,lissencephaly, Thin corpus callosum, Dysmyelination, Enlarged ventricles and Subarachnoid space	
Severe microcephaly	f	30‐33GW	c.1735G>A (p. Gly579Arg), c.1358 dup (p. Tyr453*)	1	Extreme microcephaly, Delayed primary gyration, Thin corpus callosum	
Severe microcephaly	f	30‐33GW	c.1735G>A (p. Gly579Arg), c.1358 dup (p. Tyr453*)	1	Extreme microcephaly, Delayed primary gyration, Thin corpus callosum	
Severe microcephaly	f	6–17 yrs	c.3820_3835del (p. Pro1274 Thrfs*56), c.5453G4 T (p. Gly1818Val)	3,9	Extreme microcephaly, Spastic tetraplegia, Seizure, Scoliosis, Precocious puberty, Cortical atrophy, Thin corpus callosum, Cerebellar atrophy, Dysmyelination	
HYC3	m	16GW	c.146_147insG (p. Thr52 fs), c.673delC (p. Leu225 fs)	1	INF, Hydrocephalus, VSD, Fetal hydrocephalus, Cleft lip and palate, Hydrops fetalis, Hepatomegaly and Cerebellar hypoplasia	Recently study

HYC3: Hydrocephalus, congenital, 3, with brain anomalies (MIM 17967). CAMRQ2: Cerebellar ataxia, mental retardation, and dysequilibrium syndrome 2 (MIM 610185).

F, female; GW, gestation weeks; m, male; M, month; NA, not available; INF, increased nuchal fold thickness; VSD, ventricular septal defect.

**FIGURE 3:**
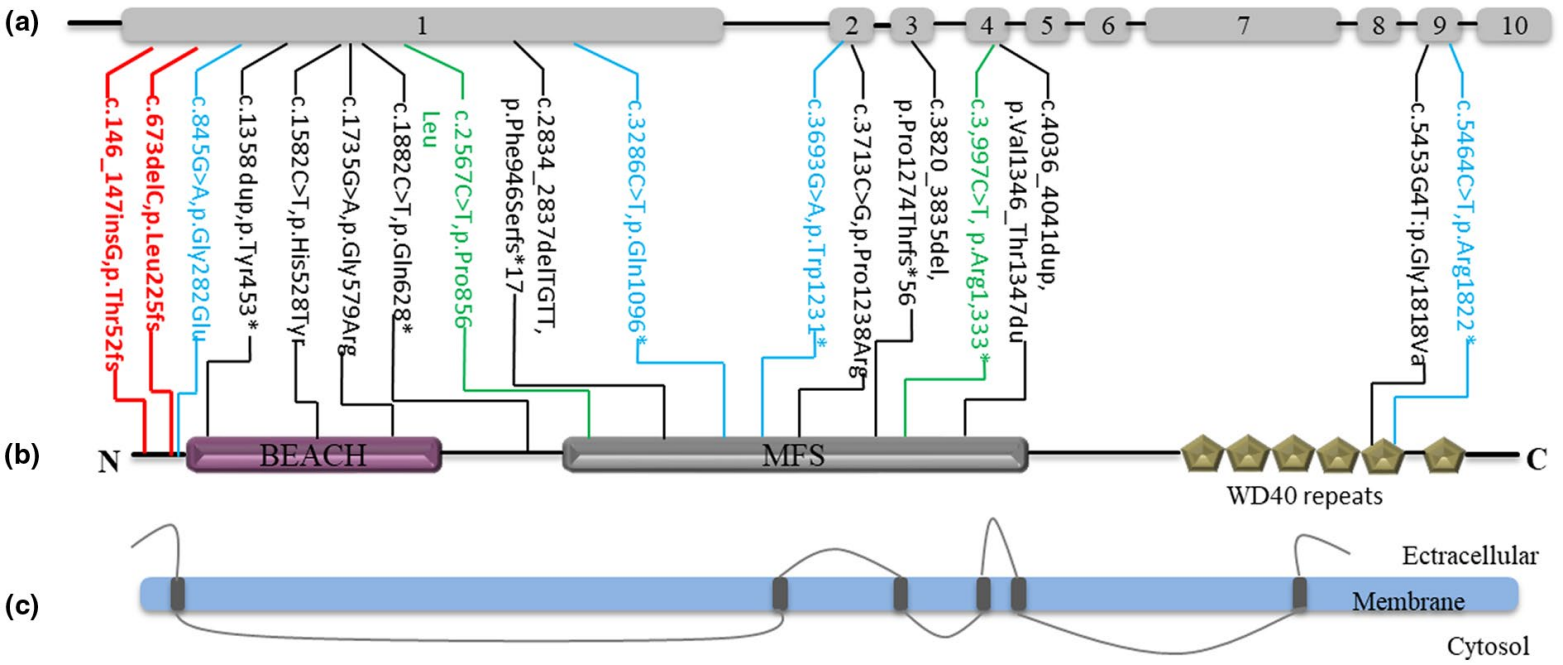
Schematic representation of the gene *WDR81* and variants associated with neurological phenotype. (a) Genomic organization of the human *WDR81* gene, predicted domain structure of the protein (b) and transmembrane domains (c), the mutations reported in previously studies were plotted with HYC3 patients (blue), CAMRQ2 patients (green) and severe microcephaly patients (black) (Alazami et al., 2015; Cappuccio et al., 2017; Cavallin et al., 2017; Gulsuner et al., 2011; Komara et al., 2016; Shaheen & Alkuraya, 2017). The variant reported in this study was indicated in red

Next, we summarized the pathogenic and likely pathogenic *WDR81* variants that have been identified, a total of 15 variants were included (Figure 3). The distribution of variants in *WDR81* and functional regions of the protein were modified to analyze the relationship between genotype and phenotype (Figure 1). Of note, Shaheen et al. identified two mutations including a truncating mutation (c.3286C>T, p. Gln1096*) and a missense variant (c.845G>A, p. Gly282Glu) in two families with severe congenital hydrocephalus. The proband with mutation c.3286.C > T (p. Gln1096*) shown severe hydrocephalus and hypoplastic cerebellum, similar presentation was found in a male neonate with a homozygous missense (c.845G>A, p. Gly282Glu), which suffering from Dandy Walker malformation with severe hydrocephalus and brain atrophy (Shaheen et al., 2017). Cappuccio and colleagues reported a patient with two in trans nonsense alleles (c.3693G>A, p. Trp1231*, c.5464C>T, p. Arg1822*) in *WDR81*, the patient presented cerebral manifestations (corpus callosum, enlarged third ventricle, cerebellar hypoplasia and extremely thin frontal lobes) and facial dysmorphism (ocular proptosis, thin upper lip, bulbous nose, mid‐face hypoplasia, and macroglossia) (Cappuccio et al., 2017). Alazami et al. identified a case carried the homozygous mutation of c.845G>A (Gly282Glu) presented with neonatal death due to severe hydranencephaly and cerebellar hypoplasia and the initial phenotype was CAMRQ2 (Alazami et al., 2015), this case was uncover the same mutation and the phenotype was similar to one of the case reported by Shaheen et al. Mutation of *WDR81* also proposed associated with sever microcephaly. Five compound heterozygous mutations in seven patients identified by Cavallin et al. shared the common phenotype of microcephaly and cerebral manifestations (Table 1). Including our case, variants lie in exon 1 with alternatively transcript isoform (NM_001163809.1, NP_001157281.1) presented most frequently (58%, 10/17), however, no significant correlation was found among variant type, variant position, protein domain, and patient phenotype due to the limited cases.

The underlying mechanism of *WDR81* caused neurological diseases still need to elucidate. Several studies have revealed that *WDR81* was particular highly express in cerebellum and corpus callosum of human brain (Cavallin et al., 2017) and increased expression in purkinje cell layer in mouse cerebellum (Gulsuner et al., 2011), Wang et al. revealed that *WDR81* deficiency leading the disruption of endosomal phosphatidyl inositol phosphate 3‐dependent endosome conversion through the TGFβ signaling pathway and negatively regulate neurogenesis in mice adult neural progenitor cell (Wang et al., 2018), similar finding with Liu et al. and Rapiteanu et al. (Liu et al., 2016; Rapiteanu et al., 2016). Cavallin et al. showed that *WDR81* disruption was associated with an increased mitotic index and delayed prometaphase/metaphase transition, however, they did not find the defects of early and late endosomes in *WDR81* patient's fibroblasts and Drosophila neural stem cells with *WDR81* knockdown (Cavallin et al., 2017), indicating that the expression for *WDR81* maybe differential in variable cells or tissues. Traka et al. performed the N‐ethyl‐N‐nitrosourea‐induced mouse suggesting that a *WDR81* missense mutation, Leu1349Pro, causes adult‐onset and progressive Purkinje cell death as well as early‐onset photoreceptor cell loss (Traka et al., 2013), however, the mechanism of pathology of *WDR81* mutation‐related phenotype was unclear.

In conclusion, based on the clinical presentations and genetic findings, we proposed that our patient's phenotype is consistent with autosomal recessive HYC3, and the condition was due to the compound heterozygous frameshift variants in *WDR81*, c.146_147insG and c.673delC in exon 1 affected N‐domain of *WDR81* protein. This was the first Chinese case with HYC3 reported to our knowledge, which helped to expands the mutation spectrum of *WDR81* and further define the genotype–phenotype correlations of HYC3. We suggested fetal hydrocephalus with extracerebral manifestations may be suggestive of *WDR81* or other hydrocephalus‐related genes deficiency, and WES should be triggered for achieving a diagnosis.

## CONFLICT OF INTERESTS

The authors declare that they have no competing interests.

## AUTHORS’ CONTRIBUTIONS

Jiasun Su and Zailong Qin wrote the manuscript, conceived and designed the experiments. Zailong Qin, Mengting Li, Qiang Zhang, Fei Chen, Qi Yang, Sheng Yi, Limei Huang and Xunzhao Zhou performed the experiments. Shang Yi and Weiliang Lu contributed to data analysis. Yingping Shen and Jingsi Luo helped to revise the manuscript.

## Data Availability

The data that support the findings of this study are available from the corresponding author upon reasonable request.
